# Savings for resilience: Investigating saving instruments in Mali

**DOI:** 10.1371/journal.pone.0326873

**Published:** 2025-07-11

**Authors:** Annkathrin Wahbi, Eike Nordmeyer, Tim Ölkers, Oliver Musshoff

**Affiliations:** Department of Agricultural Economics and Rural Development, Georg-August-University Goettingen, Goettingen, Germany; Department of Agriculture, Environment and Rural Affairs, UNITED KINGDOM OF GREAT BRITAIN AND NORTHERN IRELAND

## Abstract

Despite worldwide initiatives to alleviate poverty, 35% of Sub-Saharan Africa’s population continues to live below the poverty line. In light of this, many regard the promotion of saving as a cost-efficient and low-risk strategy for household resilience and pro-poor development. We assess saving determinants for 374 Malian farmers by employing a two-step selection model. As a first step, we assess determinants of whether or not a farmer saves by applying a probit model. In a second step, we estimate an Ordinary Least Squares regression to investigate a farmer’s savings amount. In both steps, we disaggregate the outcome variable on whether respondents save through mobile money, via a bank account, or a secret place. To address endogeneity concerns, we apply an instrumental variable approach using the walking distance to the next mobile money agent as an instrument. We find considerable heterogeneity in saving determinants and identify a particularly strong role of supply-side factors such as infrastructure quality. Furthermore, the results suggest that saving with a secret place is persistently popular, in particular among younger respondents and those who do not have access to a smartphone in their household. This indicates a potential to transfer these hidden savings to formal accounts for interest earnings and potentially safer storage. The findings have implications for improving financial practices and resilience among smallholder farmers in low-income economies, suggesting the transformative potential of secure and accessible saving mechanisms.

## 1. Introduction

Despite global efforts to alleviate poverty, 35% of the Sub-Saharan African (SSA) population still lives on less than $2.15 per day [1]. One avenue to tackle persistent poverty is microfinance, whereby marginalized households get the chance to invest, protect themselves against shocks and smooth consumption through loans, insurance or savings accounts [2,3]. In recent years, saving and household financial resource management have been identified by researchers and policy decision-makers as promising instruments for pro-poor development [4,5]. In comparison to credit or insurance, for saving, funding requirements through external capital are negligible [5,6] and there is no risk of over-indebtedness and being blacklisted [7].

Saving can, on a psycho-social level, not only increase agency and feelings of self-worth instead of dependency [8], but also enable an individual to engage in profitable agricultural investment opportunities, such as fertilizer or small machinery, and thus mitigate the impacts of adverse household shocks [5,6,9]. The latter is particularly important in the context of rural households that are often dependent on agriculture. Rural households are especially vulnerable to food insecurity because farming incomes are volatile and depend on the climate [10]. When hardships arise, these households might have no other choice but to sell property or take up loans under unfavorable conditions.

In contrast to more informal saving mechanisms, formal and semi-formal saving solutions in the form of bank accounts or mobile money (MM) accounts are usually held by an individual and are password-protected, thus creating a higher level of privacy and safety. Evidence further suggests that more formalized saving channels allow for better cash accumulation when compared to foregoing their use [11,12]. Yet, they often come with a cost, either directly in the form of account opening and running fees, or indirectly as opportunity and travel costs since Automated Teller Machines (ATMs) and MM agents might not always be close to the individual. Compared to traditional banks, however, mobile financial services are at an advantage as their technology is accessible through the oftentimes more widespread agent network [13]. Furthermore, it is a relatively cheap and easy way of storing cash on the phone. One study in Mali shows that saving with MM statistically significantly reduces the probability of temptation spending, while saving with family, friends, or at home increases the probability if such undesired spending [14].

In the past decade, a growing body of literature has contributed to the recognition of saving promotion as a sound policy measure. Studies in Kenya [11,15], Mali [16], Mozambique [17], and Uganda [18] demonstrate the beneficial effects of the provision of safe and accessible saving channels. The association of separate weather insurance, saving, and credit to individual welfare has also been examined in West Africa [19]. According to the simulation results, saving leads to substantial gains, which are higher than those achievable with unsubsidized weather insurance.

Farmers’ saving determinants have been previously investigated for example, in Ghana [20] and in Kenya [21]. One study uses a two-step or double hurdle model to assess the determinants for Ethiopian agro-pastoralists for (1) the decision or probability of saving, and (2) the accumulated amount [22]. Yet, previous studies did not differentiate between different saving tools that are available to farmers and thus are limited in what they can elicit about different effects across saving channels. Contrary to the study in Ethiopia [22], we assess the saving behavior of arable farmers. Most of our respondents cultivate cash crops (such as cereals or maize) and are, therefore, expected to have some proceeds from sales that they want to save. In contrast to subsistence farmers, they also might require lump sums for seasonal investments into inputs like fertilizer or improved seeds. Our approach is also somewhat related to a study on credit sources of Indonesian farmers [23]. Yet, while the authors look at lenders with differing degrees of formality from banks over farmer associations to agricultural input kiosks and traders, we investigate different saving channels.

The question remains as to which factors influence (i) the probability of saving as a behavior and (ii) the amount saved. Thus, in this paper, we set out to investigate this issue and, as recommended in the conclusion of [22], fill a literature gap by further disaggregating the saving channels: We analyze the factors influencing the saving probability and the associated amount concerning saving via (a) MM, (b) bank, or (c) a secret place. In the questionnaire, the option secret place was framed as ’saved with a secret hiding place’. Saving via MM, bank or secret place were the most commonly used channels. Other saving mechanisms we included in the survey were saving with a saving club (e.g. Tontine) or saving with a trusted person. The respondents could also indicate ’other’. Yet, these options were only chosen by few (N *<* 30), so we did not include them in the comparison.

To answer our research questions, we analyze a primary data set from Malian smallholder farmers using a two-step selection model by applying a probit model and subsequently an Ordinary Least Squares (OLS) regression. We apply an instrumental variable (IV) to reduce endogeneity concerns of the two-staged approach. The self-reported distance to a MM agent is used as an instrument.

Mali is an ideal setting for our research on farming households, since 80% of its population work in agriculture [24], a sector characterized by high risk of income volatility and adverse shocks. A large portion (45%) of the Malian population lives below the national poverty line [25]. Political instability in recent years has further aggravated poverty and reduced planning horizons. Furthermore, Mali has a thriving market for mobile financial services and the mobile phones needed for MM are ubiquitous. According to the International Telecommunication Union (ITU), as of 2015, 85% of the population had access to 2G and 10% had access to 3G connectivity. In the past years, mobile network technologies expanded quickly over the region and as of 2022, 70% of the population had 3G connectivity and 100% at least 2G [26]. Thus, we anticipate that the results of this paper will contribute to improving financial practices and foster greater accessibility to saving services.

We are motivated by previous research which suggests that, if implemented carefully, responsible financial resource management and saving can prove transformative for poor households [5]. Understanding the determinants of farmers’ saving decisions when using various channels is crucial to adequately advise MM providers and banks on how to increase the attractiveness of formal and semi-formal accounts to farmers and policymakers. Upon access to secure and reliable saving channels, these households could enter a virtuous cycle of saving, productivity-enhancing investment, and financial resilience [6,11]. Therefore, this study investigates the determinants of a farmer’s saving probabilities and the savings amount for different mechanisms.

Our contribution to the body of evidence is threefold: First, we show initial insights into the heterogeneous associations of relevant determinants to farmers’ saving probability for different saving mechanisms. Second, we observe that better bank infrastructure (measured in walking distance to the next bank branch) seems to improve the likelihood of higher savings. Third, and in line with the results from Ethiopia [9], we find that saving in a secret place is still a relevant saving channel. Despite the apparent ubiquity of MM, people nonetheless choose to keep a large portion of their savings at home, i.e. in a secret place. Those who are more likely to save in a secret place are, on average, younger. Furthermore, farmers who are more likely to save via a secret place have, on average, a lower probability of having a smartphone in their household. Here, we identify a considerable potential for formalizing these savings and facilitating saving behavior through increased safety, commitment-increasing behavioral mechanisms, as well as through interest earnings. The use of the IV represents a key methodological contribution by addressing endogeneity concerns. It also strengthens the robustness and internal validity of the two-stage approach. More specifically, we identify the self-reported distance to a MM agent as an appropriate IV for examining mobile money savings because it is likely to influence the probability of using mobile money services, but it does not directly affect saving behavior, except through mobile money usage. With our results we address practitioners and policy decision makers. We clearly identify a positive effect of MM on savings accumulation and advise mobile network operators and banks to further strengthen their collaboration in the field of mobile solutions and to reach out to more marginalized areas.

The paper proceeds as follows: Section 2 introduces the data and section 3 details the methodology. In section 4, we present our results and discuss them. Section 5 summarizes and focuses on concluding remarks.

## 2. Data

In the first part (section 2.1), we introduce how we collected the data. Next, we present the descriptive statistics in section 2.2.

### 2.1 Description of the data collection

**Fig 1:**
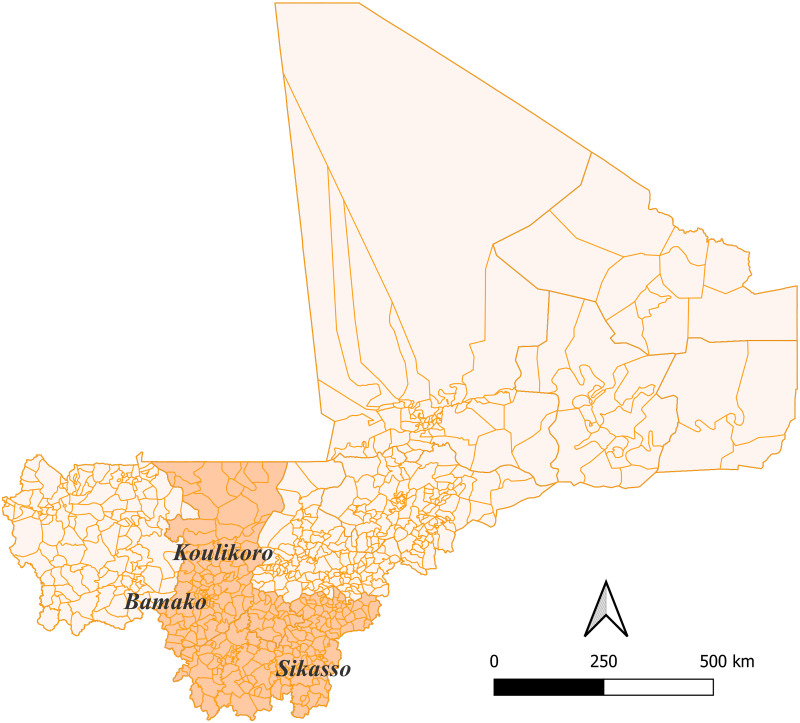
Map of Mali, highlighting the provinces where the data collection took place. Source: Own illustration.

All participants of this study are clients of the Malian bank Banque Nationale de Développement Agricole (BNDA). The BNDA is a major commercial bank operating across all sectors in Mali and Western Africa. The smallholder farmers participating in our study reside in Koulikoro (50%), Sikasso (46%), and Bamako (4%) (compare Fig 1).

The survey took place in the post-harvest season from December 2022 to February 2023. For this research, ethical approval was obtained by the German Institute of Development and Sustainability (IDOS). We have furthermore obtained clearance from the ethics board of the University of Göttingen, Germany. Verbal informed consent was obtained, and confidentiality of the information was secured by excluding respondents’ identifiers, such as names, from the data collection format. We did not interview minors.

In November 2022, we conducted a one-week training session in Bamako, focusing on thoroughly preparing enumerators to minimize potential interviewer biases. Enumerators were compensated for both their participation in data collection and the in-person training week. Our local partners assisted in sourcing enumerators, all of whom had prior experience in data collection. They further recommended placing an emphasis on training enumerators not only in French, but also in Bambara. This additional language training was deemed crucial, considering that many respondents, particularly those in rural areas, might prefer Bambara over French. Eventually, most interviews were conducted in Bambara. To target lower-income individuals, we only interviewed clients of the microfinance arm of BNDA. We then randomly selected individuals from a list provided by BNDA staff. All survey participants were interviewed face-to-face at their homes by our enumerators. After a completed interview, the respondents received non-monetary compensation. The enumerators did not observe any indications of bias, such as elevated drop-out rates or patterns of socially desirable responses. We aimed for a sample size of 400; subsequently interviewing 405 smallholder farmers. After data cleaning to remove incomplete questionnaires, 374 observations remained to constitute the final sample size. The code book can be found in S1 Table in the Supporting Information.

### 2.2 Descriptive statistics

Table 1 shows the socioeconomic characteristics of the sampled farmers. The mean age of the farmers is 47 years and the majority are male (95%). Mali’s society is characterized by male household authority figures and patriarchal lineages [27]. Thus, the male household head is most likely the authority signing credit contracts. As we interviewed bank clients, we expected a high share of male participants. Most of the sampled farmers have no formal education (59%), while 41% attended at least primary school. Additionally, 33% can speak, read, and write in French, which we use as a proxy for education. Most of the farmers describe themselves as ethnic Bambara (59%). We observe that the average household size is 12 and the number of wives per household varies between 0 and 5. The average savings amount per farmer at the time of the survey is 846,759 F CFA (approximately 1,264 Euro). Some, 29%, of the farmers also have a job outside the agricultural sector and 33% stated that they have migrated for work-related reasons. At the time of the survey, walking to the closest bank branch was possible for 19% of the respondents, while 58% lived within walking distance to a MM agent. The farmers stated having to walk, on average, 10 minutes to access a MM agent and 30 minutes to access the next bank. The number of plots varies between 0 and 13, while the number of crops also varies between 0 and 13. The 9 farmers that stated that they cultivate zero crops are livestock farmers.

**Table 1:** Summary statistic.

	Mean	SD	Min	Max
**Farmer’s characteristics**				
Farmer’s age, in years	47.29	10.00	24	80
Dummy if farmer is male (0: no, 1: yes)	0.95	-	0	1
Education Dummy (0: no, 1: yes)				
Dummy if farmer has at least some primary formal education	0.41	-	0	1
Dummy if farmer has writing skills in French	0.33	-	0	1
Dummy if farmer has some oral French skills	0.33	-	0	1
Dummy if farmer’s ethnicity is Bambara (0: no, 1: yes)	0.59	-	0	1
Farmer’s number of wives	1.25	1.14	0	5
Dummy if farmer has a job outside of agricultural sector (0: no, 1: yes)	0.29	-	0	1
Dummy if farmer has migrated for work (0: no, 1: yes)	0.33	-	0	1
Farmer’s total savings at the time of the survey (in F CFA^a^)	846,759	2,642,643	0	30,000,000
**Household characteristics**				
Farmer’s household size (continuous variable)	12.21	7.25	1	37
Farmer’s region (categorical variable)	2.93	1.03	1	4
Dummy if household has any phone (0: no, 1: yes)	0.99	-	0	1
Dummy if household has a smartphone (0: no, 1: yes)	0.64	-	0	1
**Distances**				
Dummy if it is possible to walk to bank branch (0: no, 1: yes)	0.19	-	0	1
Minutes farmer has to walk to next bank branch	30.4	47.22	0	300
Dummy if it is possible to walk to MM agent (0: no, 1: yes)	0.58	-	0	1
Minutes farmer has to walk to next MM agent	10.02	10.70	0	60
**Agricultural characteristics**				
Farmer’s number of plots (continuous variable)	3.60	2.11	0	13
Farmer’s number of crops (continuous variable)	3.39	1.91	0	13
Observations	374			

^a^F CFA refers to the CFA-Franc (issued by the Central Bank of the West African States (BCEAO)) and is tied to the Euro with a fixed exchange rate of 655.96 F CFA = 1 €. Source: Own illustration.

In the study context, both simple phones and smartphones are suitable for MM operations. At the household level, we observe a cellular phone ownership rate of 99% and a household smartphone ownership rate of 64%. This makes the use of MM services feasible for wealthier and poorer farmers alike. All explanatory variables included in our estimation strategy, along with their coding, can be found in S2 Table in the Supporting Information.

To assess the representativeness of our results and generalize our findings to the entire Malian context, we compare the summary statistic of our sample with Mali’s representative Living Standards Measurement Study (LSMS) data [28] in S3 Table in the Supporting Information (see also [29]). Comparing the mean values for various household characteristics of our sample and the LSMS sample, we conclude that our sample is comparable to several characteristics of Malian farmers in the three respective regions.

**Fig 2:**
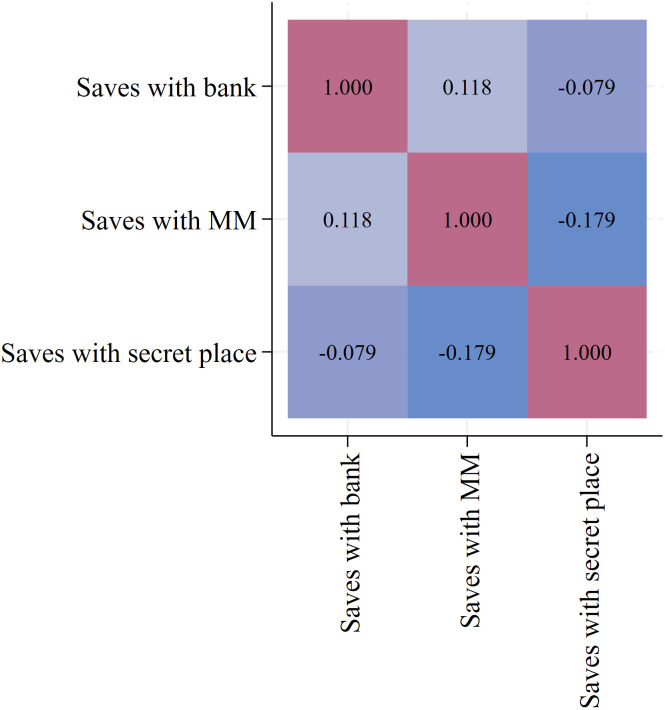
Correlation plot of three saving channels of interest. Source: Own illustration.

Fig 2 presents a correlation plot of the three dummy variables of interest: whether a farmer saves using a bank account, MM, or a secret place. The correlations between these three saving channels are low. The low correlation values, ranging between -0.079 (saves with secret place and saves with bank) and 0.118 (saves with MM and saves with bank), indicate that the three dummy variables, saving with a bank account, MM, or a secret place, are largely independent of one another. This suggests that a farmer’s choice of one saving channel does not strongly influence their likelihood of using another. In other words, these saving methods appear to cater to distinct saving behaviors or needs, rather than being substitutes or complements.

## 3. Estimation strategy

In the first part of this section (3.1), we explain our empirical model. Subsequently, we introduce an IV to test the robustness of our results and to account for potential endogeneity concerns (section 3.2).

### 3.1 Empirical model - two-part selection model

To investigate the factors influencing Malian farmers’ saving probability and their cash amount per saving channel, we apply a two-part selection model. We employ a probit regression for the probability of observing a positive-versus-zero outcome (see also [30]). Conditional on a positive outcome, we apply an OLS regression as suggested by [31], and used in previous research [32–34]. We do so as our research interest is twofold: First, we are interested in the determinants of a farmer saving with different channels, and second, we aim to investigate the respective savings amount. In addition, a considerable share of our dependent variables (total household savings and savings per channel) are zero values, as shown in S2 Table in the Supporting Information. By omitting these households from the analysis, the population sample might not be random, as it does not include potential savers. In general, various approaches have been developed and employed to deal with a considerable number of zero values (e.g. Heckman selection model, tobit or poisson [35]).

By comparing the two-part selection model with the Heckman selection model, important differences become evident. First, despite their initial similarity, the two-part model should not be viewed as being nested within the Heckman selection model and equivalent when there is no selection on unobservables. The two-part model does not make any assumption about the correlation between the errors of the binary and continuous equations. Second, from a conceptual standpoint, the zeros in the Heckman selection model denote censored values of the positive outcome, while zeros in the two-part model are true zeros. Finally, Monte Carlo evidence shows that when the data are generated from the generalized tobit model without exclusion restrictions to identify the “zeros” equation, the two-part model generally produces better estimates of the conditional mean and of marginal effects (ME) than the correctly specified generalized tobit model [31].

Our two-part selection model is described subsequently. To model whether or not a farmer is saving with the saving channels, we use a probit model. The latent variables contain the probability of observing any savings per channel, which is defined by:


$$y_{i}\;=\;\beta_{i}X_{i}+\varepsilon_{i}$$ (1)

$$y_{i}=\left\{ \begin{array}{c} 1\;if\;y_{i}^{*}>0\;\\ 0\;if\;otherwise \end{array}\right.\;$$ (2)

Where $y_{i}^{*}$ is a latent dummy variable having the value of 1 if the farmer saves with the respective channel and has the value of 0 when the farmer does not report savings (Where $y_{i}^{*}$ = 0). The parameter vector containing the coefficients of the selection equation is represented as *β*_*i*_, *x*_*i*_ is a vector of the set of explanatory variables, the subscript *i* indicates a farmer’s observation, and *ε*_*i*_ describes the error term. We apply heteroskedasticity robust standard errors.

Following the literature [36–38], we included a set of socio-demographic variables (age, education, ethnicity, gender, mobile phone ownership, number of wives) as explanatory variables in the probit model. Furthermore, we control for the number of savings accounts, the possibility to walk to a bank branch as well as to a MM agent. Finally, a farmer’s risk perception of having money stolen was investigated. All explanatory variables that were included in our estimation strategy and the vector *x*_*i*_ as well as their codings can be found in S2 Table.

In the probit model, the amount of savings is not included. Therefore, to model the reported savings amount by farmers in general, and with respect to the different channels, we estimate an OLS regression after the sample selection given that savings are reported as a continuous outcome. Accordingly, a farmer’s expected savings amount is defined as follows:


$$y_{i}\;=\;\beta_{i}X_{i}+\varepsilon_{i}$$ (3)

where *y*_*i*_ denotes the respective dependent variable that indicates the amount of savings of a farmer *i* per channel; *x*_*i*_ is a column vector of all explanatory variables; *β*_*i*_ depicts a vector of unknown parameters; and the scalar *ε*_*i*_ represents the error term. Again, we use heteroskedasticity robust standard errors. Similar to the probit model, we investigated the same set of determinants for *x*_*i*_ in the OLS regression as the effect of these determinants might be different for the savings intensity compared to the savings decision. All included variables were examined for possible multicollinearity prior to estimation. All variance inflation factors (VIF) are below 5 and all tolerances are higher than 0.1. Thus, no multicollinearity concerns arise. In addition, the model was estimated with heteroskedasticity robust standard error [39].

### 3.2 Instrumental variable as robustness test

The IV approach is widely used to address endogeneity concerns, especially when associated with non-experimental data such as our sample selection model [40]. This issue can lead to biased and inconsistent parameter estimates, compromising the internal validity of the statistical analysis.

The IV method relies on the availability of a suitable instrument. The instrument must be a variable that is correlated with the potentially endogenous explanatory variable, but uncorrelated with the error term. In other words, the instrument affects the dependent variable only through its association to the endogenous explanatory variable [41]. We propose using the self-reported distance in minutes to the nearest bank branch and the distance to the nearest MM agent as instruments. We argue that the selected variables influence the likelihood of using saving services but do not affect the savings amount. These instrumental variables are chosen based on the plausible assumption that proximity to a bank or MM agent affects an individual’s financial behaviors and, by extension, their overall savings.

In economics, distance is a commonly used instrument. One example is the IV proximity to an educational institution as an exogenous determinant of schooling [42] with the rationale being that the proximity to educational institutions should substantially reduce the costs of attending college for school-aged children but should otherwise have no associations to education. In another study [43], inverse distance to the closest palm oil mill serves as an instrument to investigate the effects of palm oil contract farming on the diets of palm oil smallholders. In their setting, the instrument should capture the distance to the next contracting company and, therefore, the likelihood of the farmer receiving a contract offer but should otherwise have no effects on the diets of palm oil smallholders. Finally, distance to a former British base in India is employed as an instrument for investigating female empowerment and its effects on household consumption and financial decisions in a matrilineal society [44]. The historical exogenous cultural shock serves as the instrument to investigate the effect of female empowerment on household financial decision-making.

Proximity to banking facilities and MM agents could have a considerable effect on an individual’s financial behavior and saving opportunities. We expect the distance to these facilities to primarily affect overall savings through accessibility rather than directly influencing the savings amount. Distance or proximity to a financial facility is expected to be independent of unobservable characteristics that may affect overall saving (e.g., risk preferences or intrinsic saving habits). As all the respondents are rural residents, we do not anticipate rural-urban disparities to play a major role in the sample. The proposed instruments for overall savings are thus based on two proximity variables: distance to the closest bank branch and distance to the nearest MM agent.

An IV must satisfy two requirements. First, the instrument must be correlated with MM savings/bank savings, which is also known as the relevance assumption [45]. During the first stage, we therefore regress the endogenous variable (amount saved with bank account and MM) on the instrumental variables (distance to bank branch/distance to MM agent in minutes and transformed to a logarithm) and other control variables that may affect savings. The following eight control variables are included in the regression: (1) the farmer’s age, (2) gender, (3) the number of wives, (4) the dummies indicating whether a farm household has a smartphone, (5) belonging to the ethnicity Bambara, (6) having some French skills, (7) the self-assessed risk of money getting stolen when kept at home, and (8) the number of saving channels used over the past 12 months. We include the same control variables in both, the first-stage and the second-stage estimation. We estimate the IV with heteroskedasticity robust standard errors. The estimated coefficient of the distance to the bank branch in this stage reflects its association to overall savings through its effect on banking accessibility.

For the second requirement, known as the exclusion restriction, the respective saving channel must only affect overall savings through the effect of the distance to MM savings and the distance to bank savings. Because one cannot test this latter assumption, its validity should be discussed [45]. If the exclusion restriction holds, a local average treatment effect can be identified [46]. The rationale is that the expansion of the MM network and bank branches are utilized as a source of exogenous variation in access to savings. The exclusion restriction might hold because neither the distance to the nearest bank branch, nor to the nearest MM agent should have a direct association to an individual’s financial well-being. The only effect of distance is on an individual’s ability to access banking services and/or an MM agent, which in turn affects their saving behavior. Because only a share of the farmers stated that they could walk to the next bank branch, the sample size for this robustness test is reduced. Walking to a MM agent is an option for 114 respondents, while only 38 stated that they could walk to a bank branch. As the small sample size of 38 would reduce the IV’s credibility, we only use the distance to the next MM agent as the instrument.

## 4. Results and discussion

In the first part of this section (4.1) we present and discuss the results. The second subsection (4.2) includes the IV results to test the robustness of our estimations.

### 4.1 Results - two-step selection model

To assess whether different saving channels can promote the saving probability and the savings amount of Malian farmers, we employ a two-step model with two equations. With the selection equation (see equation (2)), we estimate the farmer’s use of the saving channels MM, bank, or secret place (subsection 4.1.1). Equation (3) models the factors influencing the respondent’s savings amount for the different saving accounts (subsection 4.1.2). In the following two subsections, we present and discuss the results.

#### 4.1.1 Saving probability.

According to Table 2, 306 farmers use at least one saving channel (82%). In particular, 190 farmers save via MM (51%), 119 farmers use bank accounts (32%) and 84 farmers (22%) save in a secret place. Table 2 presents the marginal effects of the probit model regarding a farmer’s probability of saving with the different saving channels. We are aware of the ongoing debate regarding the use of p-values and statistical inference in scientific research [47,48]. Despite this discussion, we use statistical significance measures in our analysis to interpret differences between coefficients, as they are widely accepted in our field and provide a standardized method for assessing evidence against the null hypothesis. At the same time, we consider it essential to not only focus on statistically significant results but also discuss effect sizes and unexpected effects.

**Table 2:** Marginal effects of the probit model for a farmer’s saving probability.

	(1)	(2)	(3)	(4)
Independent variable	Total savings	MM savings	Bank savings	Secret place savings
Farmer’s age, in years	-0.002	0.000	0.007^***^	-0.005^**^
	(0.002)	(0.002)	(0.002)	(0.002)
Dummy if farmer is male (0: no, 1: yes)	0.055	0.152	0.184^*^	-0.075
	(0.089)	(0.116)	(0.102)	(0.067)
Dummy if farmer has some French writing skills (0: no, 1: yes)	0.056	0.084	0.041	-0.025
	(0.042)	(0.052)	(0.040)	(0.042)
Dummy if farmer’s ethnicity is Bambara (0: no, 1: yes)	0.060	0.180^***^	-0.025	-0.026
	(0.043)	(0.052)	(0.041)	(0.040)
Farmer’s number of wives	0.009	-0.017	0.003	0.015
	(0.019)	(0.021)	(0.016)	(0.015)
Total number of farmer’s savings accounts	0.284^***^	0.303^***^	0.316^***^	0.223^***^
	(0.040)	(0.048)	(0.025)	(0.024)
Dummy if it is possible to walk to bank branch (0: no, 1: yes)	0.096	-0.095	0.153^***^	0.026
	(0.064)	(0.066)	(0.051)	(0.050)
Dummy if it is possible to walk to MM agent (0: no, 1: yes)	0.087^**^	-0.026	0.073^*^	0.025
	(0.039)	(0.052)	(0.042)	(0.041)
Dummy if farmer’s household has a smartphone (0: no, 1: yes)	0.006	0.063	0.067	-0.132^***^
	(0.038)	(0.047)	(0.041)	(0.039)
Self-assessment risk of getting money stolen when keeping it at home	-0.006	0.018	0.013	-0.036^***^
	(0.013)	(0.016)	(0.012)	(0.014)
Regional fixed effects	Yes	Yes	Yes	Yes
Pseudo R^2^	0.218	0.169	0.329	0.231
Savings reported	306	190	119	84
Observations	374	374	374	347

The total savings amount as well as the amounts for the different saving channels were transformed to logarithmic outcomes (see column (1) to (4)).

Heteroskedasticity robust standard errors in parentheses.

* *p <* 0.10, ^**^ *p <* 0.05, ^***^ *p <* 0.01.

The description of the variables can be found in S2 Table in the Supporting Information.

Source: Own illustration.

Our results suggest that older farmers have a statistically significant higher probability of saving with a bank account. In contrast, a statistically significant negative association of age to the likelihood of saving in a secret place is identified. According to the ME, the probability of saving at a bank increases by 0.7% per additional year of age, while the probability of saving in a secret place decreases by 0.5% per year. The magnitude of these coefficients is rather small. We find a slightly positive, but statistically insignificant association of age with respect to saving with MM.

Compared to females, male farmers have a higher probability of saving with MM and at a bank, while they have a lower probability of saving in a secret place. In particular, the probability of saving with MM is 15.2% for male farmers. Furthermore, the probability of saving at a bank is statistically significantly higher for male farmers by 18.4%. The latter finding is somewhat intuitive given that the literature indicates that women might feel particularly pressured to share their income with kin to avoid social consequences [6,49,50]. Considering this, saving in a secret place could be attractive from a woman’s perspective. However, based on our data we cannot say whether women genuinely prefer to save money in a secret place, or if they do so out of necessity due to the lack of accessible and secure alternatives, such as bank accounts. This behavior may be attributed to their engagement in domestic tasks, rendering them less mobile and unable to access traditional banking services. Yet, our findings are not statistically significant at conventional levels and rely on a small fraction of female respondents (N=20).

Further, the farmer’s education, measured as being literate in French, is positively, however not statistically significantly, associated with saving probability in general and saving with MM and at a bank in particular. In contrast, French literacy is negatively associated with saving money in a secret place. Education is often considered a key determinant of technology adoption such as MM [51,52]. Even though our results indicate this is also true for Malian farmers, the values are not statistically significant at conventional levels.

Farmers identifying themselves as Bambara have a statistically significantly higher probability of saving with MM by 18.0%. They further have a 2.5% lower probability of saving money at a bank, and a 2.6% lower probability of saving in a secret place, however this is not statistically significant. While the variable number of wives is not statistically significantly associated to any of the dependent variables, the number of savings accounts shows a statistically significant positive association to all saving channels, indicating that our results are reliable.

Our results show that the possibility of walking to a bank has a statistically significant positive association with the likelihood of saving at a bank. In particular, the probability of saving money in a bank account increases by 15.3%. Therefore, in cases where several opportunities exist, saving with a bank account seems to be attractive to farmers. The possibility of walking to a MM agent has a statistically significant positive association to saving in general and to saving at a bank. More specifically, the probability of saving money increases by 8.7% in general and the probability of bank savings by 7.3%. Surprisingly, a slightly negative, though not statistically significant association was found for saving with MM.

Owning a smartphone has a slightly positive but not statistically significant association with a farmer’s probability of saving money with MM or at a bank. On the contrary, it has a statistically significant negative association with the probability of saving money in a secret place. More specifically, farmers with access to a smartphone have a 13.2% lower probability of saving money in a secret place. This could indicate that once savers have the opportunity to deposit their cash formally in a bank account or semi-formally via MM, they prefer this over keeping it in a secret place where it might be stolen or spent more easily.

Lastly, we asked the farmers to rate the risk of getting money stolen on a scale from very low to very high on a 5-point equally spaced Likert scale. We find that perceiving one’s own financial situation as risky has a slightly positive, however, not statistically significant positive association on farmers likelihood to save money with MM or at a bank. On the other hand, a lower risk perception is associated with saving in a secret place in a statistically significant negative way. The marginal effect indicates that a one-point higher risk perception decreases the probability of saving in a secret place by 3.6%.

#### 4.1.2 Savings amount.

Table 3 shows the results of the OLS regression after the sample selection. All independent variables of the probit model were included in the OLS regression. While the results are mostly similar in terms of sign, magnitude, and statistical significance, some variables show different associations. Therefore, the differences are of major interest in this section. To increase the model performance and allow the coefficients to be interpreted as direct elasticities, the total savings amount as well as the amounts for the different saving channels were transformed to logarithmic outcomes.

**Table 3:** Results of the OLS regression regarding the determinants of a farmer’s savings amount.

	(1)	(2)	(3)	(4)
Independent variable	Total savings	MM savings	Bank savings	Secret place savings
Farmer’s age, in years	0.019^*^	0.018	0.119^***^	-0.069^**^
	(0.010)	(0.029)	(0.029)	(0.028)
Dummy if farmer is male (0: no, 1: yes)	0.513	1.747	3.109^*^	-0.908
	(0.330)	(1.451)	(1.608)	(1.118)
Dummy if farmer has some French writing skills (0: no, 1: yes)	0.020	0.471	0.507	-0.461
	(0.194)	(0.631)	(0.675)	(0.584)
Dummy if farmer’s ethnicity is Bambara (0: no, 1: yes)	-0.289	1.903^***^	-0.838	-0.666
	(0.203)	(0.683)	(0.747)	(0.623)
Farmer’s number of wives	0.019	-0.453	-0.049	0.268
	(0.074)	(0.282)	(0.293)	(0.258)
Total number of farmer’s savings accounts	1.095^***^	2.277^***^	4.299^***^	3.071^***^
	(0.120)	(0.436)	(0.413)	(0.380)
Dummy if it is possible to walk to bank branch (0: no, 1: yes)	0.890^***^	-1.208	2.614^***^	0.281
	(0.237)	(0.804)	(0.875)	(0.754)
Dummy if it is possible to walk to MM agent (0: no, 1: yes)	0.127	-1.061	0.754	0.309
	(0.195)	(0.665)	(0.701)	(0.599)
Dummy if farmer’s household has a smartphone (0: no, 1: yes)	0.632^***^	0.943	1.503^**^	-1.600^**^
	(0.196)	(0.621)	(0.711)	(0.622)
Self-assessment risk of getting money stolen when keeping it at home	0.087	0.412^**^	0.238	-0.404^**^
	(0.062)	(0.201)	(0.221)	(0.175)
Regional fixed effects	Yes	Yes	Yes	Yes
R^2^	0.360	0.187	0.353	0.228
Observations	305	190	119	81

The total savings amount as well as the amounts for the different saving channels were transformed to logarithmic outcomes (see column (1) to (4).

Heteroskedasticity robust standard errors in parentheses.

* *p <.*10, ^**^ *p <.*05, ^***^ *p <.*01.

The description of the variables can be found in S2 Table in the Supporting Information.

Source: Own illustration.

Our results indicate that the farmer’s age has a statistically significant positive association with overall savings, and the amount of bank savings. Being one year older increases the bank savings amount by 11.9%. On the contrary, a statistically significant negative association to a farmer’s savings amount in a secret place was found. Being one year older decreases the amount of secret place savings by 6.9%. The small positive association of age with the farmer’s savings amount with MM is not statistically significant at conventional levels.

According to Table 3, compared to women, men save more with a bank account and MM but less in a secret place. Yet, the association is only statistically significant at conventional levels for the bank savings: Being a male farmer increases the amount of these savings in a statistically significant way by 310.9%. This may be explained by the disproportionate distribution of capital and the control over household finances. Since women have lower access to productive resources and capital and are often more likely to be expected to share their money with others [49], they consequently might save smaller amounts compared to their male counterparts [49,53].

French literacy is positively but not statistically significantly associated with the farmer’s total savings as well as with MM savings and bank savings. For saving via a secret place, a slightly negative association was identified, again without statistical significance.

Compared to other ethnicities, Bambara farmers have statistically significantly higher savings with MM in particular: If a farmer identifies as Bambara, their MM savings are, on average, 190.3% higher than those from other ethnic groups. In contrast, their savings at a bank are lower on average compared to other ethnic groups. In addition, Bambara farmers have lower savings in a secret place. However, given that these have no statistical significance, these associations should be considered with caution and need further investigation.

The number of wives is slightly positively but not statistically significantly associated with the farmer’s total savings as well as with secret place savings. For saving via MM and with bank savings, we observe a slightly negative association, however, without statistical significance. The number of saving accounts has, as expected, a statistically significantly positive association to a farmer’s total savings as well as on all other saving channels considered in this analysis.

In contrast, when a bank is located within walking distance, bank savings are 261.4% higher. Additionally, the total amount of savings increases by 89.0%. Both associations are statistically significant at the 1% level. Our results are in line with previous findings that argue that banks prefer establishing branches in regions with better infrastructure and potentially wealthier clients [13,54]. For perspective, a bank was only within walking distance for approximately 20% of the surveyed farmers. Therefore, we assume the bank infrastructure to be of high relevance for a farmer’s saving ambitions and more so via a formal account. The association for MM savings is negative and for secret place savings it is positive. Both associations are not statistically significant at conventional levels.

We find a positive but not statistically significant association for the distance to the next MM agent on the total savings amount, bank savings and saving in a secret place. Most notably, the availability of a MM agent nearby has a slightly negative association to MM savings. While this is a surprising outcome at first, we suspect that proximity might also lower the barrier to spontaneous withdrawals. Contrarily, the further away the agent, the harder it is for the saver to liquidate their funds. This might thus function as an indirect tool to increase the saving commitment.

In the previous subsection, we identified smartphone use as a key driver for saving via the respective saving channels. Concerning the savings amount, owning a smartphone leads to statistically significantly lower savings in a secret place. In contrast, a statistically significantly positive association was found for the total savings amount and bank savings. More specifically, smartphone owners have a 63.2% higher total savings and a 150.3% higher bank savings. Lastly, smartphone owners also have higher savings with MM. However, this association is not statistically significant at conventional levels. The analysis highlights the relevance of distinguishing between the saving channels. To ensure that smartphone adopters are not different from non-adopters, we conducted a mean comparison of some socio-demographic characteristics of both groups - adopters and nonadopters. Please find the results in S4 Table of the Supporting Information. We observe no statistically significant differences between these two groups.

The higher the subjective perceived risk of money getting stolen when saving it at home, the lower the reported savings in a secret place. A one-point higher risk perception of having money stolen when keeping it at home, statistically significantly increases the savings amount with MM by 41.2%. In contrast, a one-point higher risk perception of having money stolen reduces the secret place savings amount by 40.4%. This association confirms our expectations, as farmers might consider other saving channels as a safer way to store money. This can be claimed given that a slightly positive but not statistically significant association was found for the bank savings amount.

### 4.2 Results of the instrumental variable robustness test

As introduced in subsection 3.2, we considered two variables, which are commonly used as instruments and decided to use only one variable as an instrument: the walking distance to the next MM agent, measured in minutes. The second instrument considered - the walking distance to the next bank branch - did not meet the relevance assumption. We argue that the distance to a MM agent is a valid instrument for measuring overall savings amounts because it influences access to MM services, which can facilitate saving behavior by reducing transaction costs and improving financial inclusion. Distance is exogenous to individual saving preferences, as it is typically determined by the geographic distribution of agents rather than by personal financial decisions. It affects saving behavior indirectly by shaping access to MM services, rather than through unobserved factors that might directly influence savings amounts.

We used the continuous variable as a potential instrument. Table 4 shows the results of the first- and second-stage. We observe a statistically significant association between the instrument walking minutes to the next MM agent and the endogenous variable, total savings amounts (as continuous (column (1)). However, this statistically significant correlation does not hold for the total amount saved when transformed as a logarithmic value. We note a positive and statistically insignificant association close to zero (column (2)). Nonetheless, since we observe the expected negative association in column (1), we conclude that the relevance assumption for this instrument has been met. The expected negative and statistically significant association that we observe indicates that living closer to the next MM agent statistically significantly increases, on average, the amount saved with MM savings amounts. In contrast, as the distance to a MM agent increases the amount saved using MM decreases statistically significantly. The F-Stat is below the rule of thumb value of 10, indicating a weak instrument. However, we employ the distance to the next MM agent as a robustness test.

**Table 4:** First and second stage results for the instrumental variable approach.

	First Stage	
	(1)	(2)
	MM savings	MM savings (as log)
Minutes farmer has to walk to next MM agent	-2,595.33**	0.049
	(1,029.721)	(0.036)
F-statistic	2.75	8.22
	**Second Stage**	
	Total savings	Total savings (as log)
Minutes farmer has to walk to next MM agent	13.917**	-0.605
	(7.239)	(0.902)
Control variables	Yes	Yes
Regional fixed effects	Yes	Yes
Cragg-Donald Wald F-statistic	1.72	2.18
Overidentification test of all instruments	0.00	0.00
Observations	114	114

Notes: As not all farmers can walk to a MM agent, only the sub sample of farmers who can walk to an MM agent is included in the analysis. This reduces the sample size to 114. This estimation includes the following eight control variables: (1) the farmer’s age, (2) gender, (3) the number of wives, (4) the dummies indicating whether a farm household has a smartphone, (5) belonging to the ethnicity Bambara, (6) having some French skills, (7) the self-assessed risk of money getting stolen when kept at home, and (8) the number of saving channels used over the past 12 months. As only 114 farmers can walk to a MM agent, the sample size is reduced accordingly. Source: Own illustration.

We show the second-stage results of the IV regression in Table 4 for both instruments considered. In column (1) the dependent variable is the overall savings amount as a continuous variable, while in column (2) it is the total savings amount as a logarithmic variable. The distance to the next MM agent branch is the instrument. An overidentification test is employed to assess whether the number of instruments used in the model is excessive. The null hypothesis posits that the instruments are correctly specified and not overidentifying. The test statistic equals 0, indicating that there is no evidence to suggest overidentification.

We observe a positively and statistically significant association between the instrument and the total savings amounts (as continuous (column (1)). However, this statistically significant correlation does not hold for the instrument and the total amount saved transformed as a logarithmic variable. We note a positive and statistically insignificant association close to zero (column (2)). The results of the relatively stronger instrument (column (1)) indicate that the closer the distance to the next MM agent, the lower the overall savings. This result may seem counterintuitive at first but indicates that the amount saved with MM is relatively smaller compared to the amounts saved with a bank account. Given that the amount saved with a bank account is, on average, more than 10 times larger (compare S2 Table in the Supporting Information), this result may be less surprising.

An additional possible explanation for the positive association between the distance to MM agents and total savings amounts is that households living farther from MM agents may be relying on alternative forms of savings mechanisms or financial services that are leading to increased overall savings. For example, households in remote areas might use informal savings groups, cash-based savings, or invest in physical assets such as livestock, land, or agricultural inputs, which could all contribute to a higher level of savings, independent of MM use, which we do not capture in the survey.

An example from Brazil [55], for instance, show that microcredits are not independently distributed and that the credit amounts given are influenced by spatial characteristics. Given that the sample consists of smallholder farmers, with some living in suburban areas of Bamako and the majority residing in more remote regions, this dynamic could play a role. For those in remote areas, alternative savings methods could be more common, which may explain the observed positive relationship between distance and overall savings. This hypothesis provides a potential avenue for future research, as it suggests that the association of MM to savings could vary across different geographic regions and socioeconomic conditions. Future studies could investigate the role of informal savings mechanisms or other financial services in remote areas and how they interact with access to formal MM systems.

## 5. Conclusion

Based on a comprehensive primary data set from rural Mali, this paper investigates drivers of (i) saving probability and (ii) the respective savings amounts. We add novelty by disaggregating the respondents’ main avenues of saving, looking separately at the channels (a) MM, (b) bank account, and (c) secret place. Despite the spread of formal and semi-formal saving channels in the past years, saving in secret places remains a relevant way to put aside and accumulate cash [9]. Further, our results suggest heterogeneous effects of relevant determinants on a farmer’s probability to save via different channels. In particular, and in line with another study from Uganda [56], we find that supply-side factors such as an adequate bank branch infrastructure is associated to a better accumulation of savings. The IVs emphasize the robustness of our two-stage approach, as we identify the distance to the nearest MM agents as an appropriate instrument: The closer a farmer lives to the nearest MM agent, the larger the MM savings.

Our study suggests that while poor individuals store money in a variety of ways, informal saving channels still seem to be a popular option. On the one hand, for MM providers and banks, this provides an opportunity to pull these savings out of the hidden places and generate revenues. On the other hand, individuals minimize the risk of theft and could start earning interest for their savings. However, it seems that either, these formal mechanisms are not accessible for the poor or not attractive enough. Hence, we suggest that governments and financial institutions invest in improving the physical and digital infrastructure to reach remote rural areas. This includes establishing more MM agents and banking branches in underserved regions to also reach those often excluded from the banking system such as rural women. Financial inclusion initiatives could include subsidies for infrastructure development or public-private partnerships to increase the presence of formal saving channels. Incentives to saving such as higher interest rates on small savings accounts, low-cost or no-cost account options, and reduced transaction fees for rural populations could be introduced. To encourage a shift away from secret place savings, policymakers and institutions could launch targeted educational campaigns highlighting the benefits of formal savings, such as security or interest accrual.

In conclusion, our study provides valuable insights into the relationship between saving channels and temptation spending in Mali. These findings have important implications for policymakers and financial institutions. By considering the saving channels of farmers, policymakers can design targeted and more effective policies and financial products. Finally, with our findings, we strive to improve smallholder farmers’ resilience in SSA, especially in the face of climate change, political instability, and adverse financial shocks.

Our paper contributes to a broader debate on the analysis of saving behavior in lower income economies. However, as with many studies, this paper has some limitations. As our sample consists of mostly male farmers, which are typically the decision-makers in Malian households, we encourage further research with a female or gender-balanced sample. While we acknowledge the important role of political stability for financial decision making, long-term planning and productive investments, especially in the context of agriculture, we did not account for unrest or the political context during the time of our study. We cannot rule out that the political situation influenced our respondents’ choice of saving channel. Future research could focus on the interplay of saving behavior and political stability in a country. Lastly, spatial heterogeneity among the different savings channels could be investigated to understand whether there are spillover effects among farmers selecting one savings channel on their peers. In addition, a heterogeneity analysis by urban and rural areas might yield interesting insights, as remote areas may rely more on informal savings groups or cash-based savings, which differ from saving practices in urban areas. Hereby, future research would contribute to a more heterogeneous understanding of farmers’ saving and decision-making behavior in the region.

## Supporting information

S1 Table Code book of the questionnaire items used for analysis. This table contains the code book of the questionnaire for the key items in our analysis.
(PDF)

S2 Table Description of the variables considered for the analysis. This table contains the description of the variables that we consider in our analysis.
(PDF)

S3 Table Summary statistic LSMS data and own collected data. This table contains the summary statistic of the LSMS data and compares it with our collected data.
(PDF)

S4 Table Selected characteristics of smartphone adopters and non-adopters. This table contains a comparison of the summary statistics of smartphone and adopters and non-adopters.
(PDF)
