# Supplementary material for: Savings for resilience: Investigating saving instruments in Mali
Source: PLoS One. 2025 Jul 11;20(7):e0326873. doi: 10.1371/journal.pone.0326873 (PMC12250645; doi:10.1371/journal.pone.0326873)
Supplement: S1 Table — This table contains the code book of the questionnaire for the key items in our analysis. (PDF) [file pone.0326873.s001.pdf]

Table S.1: Code book of the questionnaire items used for analysis.

Variables	Question the variable is based on	Unit of the variable
**Dependent variables**		
Total savings	This is the sum of all saving instruments a farmer use.	in F CFA
MM	Which of the following saving instruments have you used within the past 12 months?	0: no, 1: yes
Bank	Which of the following saving instruments have you used within the past 12 months?	0: no, 1: yes
Secret place	Which of the following saving instruments have you used within the past 12 months?	0: no, 1: yes
Amount (MM)	Please indicate how much money you have saved with each of the following saving instruments (if applicable)	in F CFA
Amount (bank)	Please indicate how much money you have saved with each of the following saving instruments (if applicable)	in F CFA
Amount (secret place)	Please indicate how much money you have saved with each of the following saving instruments (if applicable)	in F CFA
**Independent variables**		
Farmer's age, in years	How old are you?	years
Farmer's gender	What is your gender?	1: male, 2: female
Dummy if it is possible to walk to MM agent	How many minutes do you have to walk to reach the next mobile money agent?	0: no, 1: yes
Dummy if it is possible to walk to bank branch	How many minutes do you have to walk to reach the next bank?	0: no, 1: yes
Dummy if farmers' HH has smartphone/ any phone	Does someone in your household possess any handheld device for telephoning?	1: no phone, 2: calls only, 3: internet access, 4: calls and internet
Risk perception	If you kept the money at home how high would you rate the risk of it getting stolen?	1: very low, 2: low, 3: neutral, 4: high, 5: very high
Dummy if farmer has some writing French skills	Do you have any writing skills in French?	0: no, 1: yes
Dummy if farmer's ethnicity is Bambara	Which ethnic group are you part of?	1: Other, 2: Bambara, 3: Bobo/ Bomu, 4: Bozo/ Tyako, 5: Dogon/ Dôgôsô, 6: Khassonké/ Khassonkakan, 7: Malinké/ Maninkakan, 8: Minianka/ Mamara, 9: Peulh/ Fulfulde, 10: Samogo/ Dungooma, 11: Sarakole/ Sooninke, 13: Sénoufo/ Syenara
Total number of farmers' savings accounts	Sum of all savings accounts the farmer has	Number
Farmer's number of wives	How many wives do you have?	Number

The questionnaire was conducted in French and Bambara. To increase readability and comprehension for the international readership, we translated the items to English.
Source: Own illustration.
