# Supplementary material for: Savings for resilience: Investigating saving instruments in Mali
Source: PLoS One. 2025 Jul 11;20(7):e0326873. doi: 10.1371/journal.pone.0326873 (PMC12250645; doi:10.1371/journal.pone.0326873)
Supplement: S2 Table — This table contains the description of the variables that we consider in our analysis. (PDF) [file pone.0326873.s002.pdf]

Table S.2: Description of the variables considered for the analysis.

Variables	Units	Mean	SD
**Dependent variables**			
Total savings	0: no, 1: yes	0.82	-
MM	0: no, 1: yes	0.51	-
Bank	0: no, 1: yes	0.32	-
Secret place	0: no, 1: yes	0.22	-
Amount (MM)	in F CFA	149,702	280,287
Amount (bank)	in F CFA	2,083,193	4,183,987
Amount (secret place)	in F CFA	300,851	357,618
**Independent variables**			
Farmer's age, in years	Years	47.29	9.99
Dummy if farmer is male	0: female, 1: male	0.95	0.23
Dummy if farmer has some writing French skills	0: no, 1: yes	0.33	0.47
Dummy if farmer's ethnicity is Bambara	0: no, 1: yes	0.59	0.49
Farmer's number of wives	Number	1.25	1.14
Total number of farmers' savings accounts	Number	1.37	0.67
Dummy if one of the HH members has a smartphone	0: no, 1: yes	0.64	-
Dummy if it is possible to walk to bank branch	0: no, 1: yes	0.19	-
Dummy if it is possible to walk to MM agent	0: no, 1: yes	0.58	-
Farmers' perceived risk of money getting stolen when saving		2.19	1.52

Source: Own illustration.
