# Supplementary material for: Savings for resilience: Investigating saving instruments in Mali
Source: PLoS One. 2025 Jul 11;20(7):e0326873. doi: 10.1371/journal.pone.0326873 (PMC12250645; doi:10.1371/journal.pone.0326873)
Supplement: S3 Table — This table contains the summary statistic of the LSMS data and compares it with our collected data. (PDF) [file pone.0326873.s003.pdf]

Table S.3: Summary statistic LSMS data and own collected data.

		LSMS data		Study sample	
	Unit	Mean	SD	Mean	SD
Farmer's age	Years	32.08[2]	17.58	47.29	9.97
**Education**					
Dummy if farmer has no formal education	Dummy (0: no, 1: yes)	0.80	-	0.59	-
Dummy if farmer has some primary formal education	Dummy (0: no, 1: yes)	0.20	-	0.25	-
Dummy if farmer has some secondary formal education	Dummy (0: no, 1: yes)	0.08	-	0.15	-
Dummy if farmer went to University	Dummy (0: no, 1: yes)	0.00	-	0.01	-
Dummy if farmer has some oral French skills	Dummy (0: no, 1: yes)	0.26	-	0.33	-
Dummy if farmer has a job outside of agricultural sector	Dummy (0: no, 1: yes)	0.19	-	0.29	-
Dummy if farmer's ethnicity is Bambara	Dummy (0: no, 1: yes)	0.58	-	0.59	-
Farmer's household size	Individuals	10.82	6.89	12.21	7.25
Dummy if farmer owns a smartphone	Dummy (0: no, 1: yes)	0.63	-	0.55	-
Observations		2,645		374	

We are using the 2018/19 LSMS data set (INSTAT, 2022) which we adjusted for the regions (only Bamako, Koulikoro, Sikasso), the sector (only respondents who state being employed in the agricultural sector) and gender (only male, as the majority of our respondents are male) [2]the household heads are on average 53.26 years old.
Source: Own illustration.
