# Supplementary material for: Savings for resilience: Investigating saving instruments in Mali
Source: PLoS One. 2025 Jul 11;20(7):e0326873. doi: 10.1371/journal.pone.0326873 (PMC12250645; doi:10.1371/journal.pone.0326873)
Supplement: S4 Table — This table contains a comparison of the summary statistics of smartphone and adopters and non-adopters. (PDF) [file pone.0326873.s004.pdf]

Table S.4: Selected characteristics of smartphone adopters and non-adopters.

	(1) Adopters	(2) Non-adopters	(3) t-test
Farmer's age (in years)	48.07	46.82	1.17
Dummy if farmers is male	0.92	0.96	1.67
Dummy if farmer has no formal education (0: no, 1: yes)	0.59	0.59	0.16
Dummy if farmer's ethnicity is Bambara (0: no, 1: yes)	0.58	0.60	0.36
Farmer's HH size (continuous variable)	12.35	11.96	0.46
HH's total savings (in F CFA)	1,029,944	540,578	1.74
Observations	234	140	

Source: Own illustration.
